# Pharmacotherapeutic actions related to drug interaction alerts – a questionnaire study among Swedish hospital interns and residents in family medicine

**DOI:** 10.1007/s00228-024-03785-4

**Published:** 2024-12-16

**Authors:** Carina Tukukino, Naldy Parodi López, Johan Lönnbro, Susanna M. Wallerstedt, Staffan A. Svensson

**Affiliations:** 1 Department of Pharmacology, Sahlgrenska Academy, University of Gothenburg, Gothenburg, Sweden; 2 Department of Clinical Pharmacology, Sahlgrenska University Hospital, 413 45 Gothenburg, Sweden; 3 Department of Internal Medicine, Sahlgrenska Academy, University of Gothenburg, Gothenburg, Sweden; 4 HTA-Centrum, Sahlgrenska University Hospital, Gothenburg, Sweden; 5 Nötkärnan Bergsjön Primary Care, Gothenburg, Sweden

**Keywords:** Drug interactions, Physicians, Primary health care, Pharmacotherapeutic actions, Questionnaire, Decision support

## Abstract

**Purpose:**

To explore how hospital interns and residents specialising in family medicine act on drug interaction alerts in a specific patient case, and on interaction alerts in general.

**Methods:**

A 4-page questionnaire, including a fictional patient case (73-year-old woman; 10 drugs in the medication list triggering 11 drug interaction alerts) and questions regarding the use of interaction alerts in general, was distributed to interns and residents during educational sessions (November‒December 2023). The respondents were instructed to consider what actions they would take “a normal day at work” due to the risk of interactions between the patients’ drugs. In the general questions, the respondents were asked how often they access the detailed interaction information (from 1 = never to 5 = always) provided by the knowledge resource, in relation to the alert classification (D = clinically significant, should be avoided; C = clinically significant, can be handled by, e.g., dose adjustment).

**Results:**

The questionnaire was completed by 55 interns and 69 residents (response rate: 98%). In the patient case, the respondents acted on a median of 4 (range: 0‒8) drugs, most often concerning repaglinide (in a D interaction alert with clopidogrel; 96% of the interns and 96% of the residents suggested action), and omeprazole (in three C interaction alerts with citalopram, clopidogrel, and levothyroxine, respectively; 71% and 83% suggested action). Among the respondents who answered the questions about how often (rated 4/5) they access more detailed information about interactions, 56 (59%) did so for D versus 29 (31%) for C alerts (*P* < 0.001).

**Conclusion:**

Physicians act on drug interaction alerts selectively, and the alert classifications seem to guide how they are used.

**Supplementary Information:**

The online version contains supplementary material available at 10.1007/s00228-024-03785-4.

## Introduction

The prevalence of polypharmacy has been reported to increase over time [1], and being treated with many drugs puts patients at risk of drug interactions [2, 3]. Drug interactions are usually categorised as either pharmacokinetic (PK), mediated by effects on the absorption, distribution, metabolism, and/or excretion of other drug(s) [4], or pharmacodynamic (PD), implying that one drug influences the effect of another, i.e., not primarily mediated via changes in drug concentration [2]. Drug interactions may contribute to adverse drug reactions (ADRs) or diminished effect [5]. The clinical consequences of drug interactions depend not only on the drugs, but also on patient characteristics, including the current clinical status [6, 7]. Older people may be particularly at risk since they are more vulnerable to ADRs, for instance due to the age-related decline in physiological compensatory mechanisms [6]. In this age group, many people also have multiple or complex disorders requiring treatment with several drugs, further increasing the risk of ADRs and drug interactions [8, 9].

A substantial number of knowledge resources are available for clinical decision support concerning potentially problematic drug interactions [10–14]. In Sweden, the national interaction database Janusmed is integrated in almost all electronic health record systems and is also accessible via the internet. Interaction alerts triggered by Janusmed are classified according to their clinical significance: D = a clinically significant interaction where the recommendation is to avoid the combination; C = a clinically significant interaction that can be handled, for instance, by a dose adjustment or separated intake; B = an alert where the clinical relevance is uncertain or varies; and A = a minor interaction without clinical relevance [10]. Furthermore, the alerts are classified according to the level of documentation: 4 = controlled studies in relevant populations; 3 = studies among healthy volunteers and/or pilot studies among patients; 2 = well-documented case reports; 1 = incomplete case reports and/or in vitro studies; and 0 = extrapolation on the basis of studies with similar drugs [10].

Janusmed alerts initially present some brief information about the expected consequence of the alerted drug combination as well as a recommendation for clinical management, along with the above-mentioned classifications of clinical significance and documentation. With a single mouse click, the user can get access to more detailed information. In all public and in most private primary care centres in Region Västra Götaland, alerts classified as D, C, or B, irrespective of level of documentation, appear integrated in the health record systems, with some differences in how they are presented. In the hospital setting, alerts classified as D or C are generally presented. The level of documentation is displayed in both settings.

We have previously shown that many drug interaction alerts turn out to have been already addressed or not to be relevant for the specific patient [15]. Investigating drug interaction alerts triggered by the medication lists of 274 older patients, we found that only 35 (9%) out of 405 presented alerts merited action according to two specialist physicians in consensus [15]. In that study, the most commonly suggested actions were to switch omeprazole to pantoprazole to avoid problems related to omeprazole’s CYP2C19 inhibition [16], and to separate intake between levothyroxine and calcium or ferrous sulfate in order to avoid decreased absorption of levothyroxine [17]. Furthermore, a recent systematic review reported that 90% of interaction alerts are overridden by physicians [18]. However, little details are known about how physicians, in different stages of their career and in different settings, act on drug interaction alerts. In the present study, we aimed to explore how interns in a university hospital and resident physicians in primary care act on drug interaction alerts in a specific patient case as well as on drug interaction alerts in general.

## Methods

An anonymous questionnaire was distributed in print to pre-registration interns attending an educational day at Sahlgrenska University Hospital, Gothenburg, Sweden (December 2023), and to residents specialising in family medicine during an educational day in Borås, Region Västra Götaland, Sweden (November 2023). The questionnaire was filled in prior to a lecture concerning drug interactions and their alerts. The respondents were informed about the purpose of the study and that participation was voluntary. After completing the questionnaire, they could either choose to put it in a pile marked”research” or in the wastebin.

The questionnaire was designed by the authors and piloted by two specialists in family medicine. The questionnaire is available in Online Resource 1. It consisted of three parts. The first part concerned the respondents’ actions related to drug interaction alerts in a clinical scenario with a fictional patient case. The case described a 73-year-old woman with 10 drugs in the medication list, triggering 11 drug interaction alerts in Janusmed, one classified as D, seven as C, and three as B alerts (Table 1). The scenario was identical for both groups of respondents, except for the fact that the interns met the patient in the hospital setting where clopidogrel had been initiated the day before, whereas the residents met the patient at a follow-up visit in primary care shortly after the hospital stay where clopidogrel was added. Along with the questionnaire, each respondent received a complete printout of all available texts in Janusmed regarding the drug interaction alerts. In the first question (Q1) regarding the patient case, the respondent was instructed to decide on potential actions for each of the 10 drugs by ticking at least one of the following boxes: (i) no action, (ii) reduce dose, (iii) stop the drug, (iv) increase dose, or (v) other action; with space for comments. The context of this question was explained as “a regular day at work”, i.e., decision-making during time pressure requiring medical prioritising. In the second question (Q2), the respondent was to determine the importance of the actions suggested in Q1, using a scale with five steps, from 1 = not at all important, to 5 = very important. The option “no action” could be ticked for drugs with no action in Q1.

**Table 1:** The 10 drugs in the fictional patient case triggered 11 interaction alerts in Janusmed, presented along with type of interaction, classification of clinical significance and level of documentation as well as recommendations for clinical management

Drug pair	Classification^a^	Interaction^b^	Recommendation
Clopidogrel—repaglinide	D3	PK	Avoid the combination, consider switching to another hypoglycemic drug
Alendronic acid—calcium^c^	C3	PK	Separate intake
Calcium^c^—levothyroxine	C3	PK	Separate intake
Clopidogrel—omeprazole	C3	PK	Consider switching to a weaker CYP2C19 inhibitor like pantoprazole
Levothyroxine—omeprazole	C3	PK	Monitor TSH levels as usual, switch levothyroxine formulation to soft capsules or solution
Citalopram—omeprazole	C3	PK	Consider switching to a weaker CYP2C19 inhibitor like pantoprazole
Citalopram—hydroxyzine	C0	PD	Monitor ECG, monitor risk factors for QT prolongation, monitor laboratory values, decrease dose
Citalopram—clopidogrel	C0	PD	Monitor clinical signs for bleeding, consider adding pantoprazole, H2- antagonists or misoprostol for gastroprotection
Atorvastatin—clopidogrel	B4	PK	No dose adjustment is necessary
Levothyroxine—metformin	B4	UNK	Monitor laboratory values when starting or stopping drug treatment
Calcium^c^—omeprazole	B3	PK	Consider doubling the calcium dose

^a^Letter *(classification of clinical significance)*: *D *clinically significant interaction that should be avoided, *C* clinically significant interaction that can be managed by, e.g., a dose adjustment, *B* the clinical significance is uncertain or varies. *Number (level of documentation)*: 4 = controlled studies in relevant populations; 3 = studies with healthy volunteers, and/or pilot studies among patients; 2 = well-documented case reports; 1 = incomplete case reports and/or in vitro studies; and 0 = data from studies based on similar drugs

^b^*PK* pharmacokinetic, *PD* pharmacodynamic, *UNK* unknown

^c^The drug shown in the patient case was calcium in combination with cholecalciferol, but as the latter is irrelevant for interactions, only calcium is presented in the text and tables

The second part of the questionnaire focused on drug interaction alerts in general, and the extent to which the respondents click to access detailed information about alerted drug interactions including the background, the underlying mechanism, and cited references. In these questions, we also used a scale with five steps from 1 = never, to 5 = always, exploring if the classification of clinical significance and the level of documentation were related to extended reading. Finally, the third part of the questionnaire gathered background information about the respondent, including age, gender, and work experience.

### Analyses

Descriptive analyses were performed using SPSS for Windows, version 24.0 (IBM Corp., Armonk, NY, USA) (C.T.), and R version 4.3.1 (Foundation for Statistical Computing, Vienna, Austria) (S.A.S.). When entering data in a spreadsheet for analyses, we noted that many respondents who marked (iii) “stop the drug” or (v) “other” on Q1, wrote a free text comment about switching to another drug. Therefore, we added a (vi) “switch drug” category. Responses matching this category, according to two authors in consensus (C.T. and S.A.S.), were categorised accordingly. In Q2, if the respondent ticked “no action” for a drug and had not filled in anything for the same drug in Q1, we adjusted the Q1 response to (i) “no action”. Respondents with missing data were included in the calculation of proportions, with 55 (interns) or 69 (residents) used as denominator unless indicated otherwise. Two questions with replies ranging from 1 to 5 were dichotomized. Thus, participants responding 4 or 5 were categorised as (i) considering their action important in the patient case, and (ii) choosing to access the detailed information in the knowledge resource. Differences between interns and residents concerning actions suggested for specific drugs, as well as respondents’ reading of alert texts depending on alert classification, were examined using Fisher’s exact test.

### Ethics approval

This questionnaire study was assessed by the Swedish Ethical Review Authority. They determined that the Ethical Review Act was not applicable and had no ethical objections to the study (2023–02355-01).

## Results

A total of 55 out of 55 interns and 69 out of 71 residents participated in the study (response rate: 98%). Regarding the interns, 33% were ≥ 31 years of age, 58% were women, and 89% had completed ≤ 1 year of internship. Regarding the residents, 86% were ≥ 31 years of age, 48% were women, and 33% had completed ≤ 2 years of training in family medicine. Further characteristics are available in Online Resource 2.

Almost all respondents, 55 (100%) interns and 68 (98%) residents stated that they would perform at least one action related to the drug interaction alerts. The median number of drugs that the respondents stated they would act on was 4 (range: 0‒8). Actions related to interaction alerts most often concerned repaglinide, a drug that together with clopidogrel elicited a D alert. Omeprazole was the second most common drug acted upon. This drug was included in three C alerts, with citalopram, clopidogrel, and levothyroxine, respectively, as well as in a B alert with calcium. Hydroxyzine was the third most common drug acted upon, included in a C alert with citalopram. Many respondents considered their actions important (Table 2). For repaglinide and omeprazole, the most common action was a switch to another drug. For hydroxyzine, the most common action was either to stop the drug or to switch to another drug. All actions that were suggested for each drug, by interns and residents, are available in Online Resource 3.

**Table 2:** Number of respondents (percentage) who suggested an action for one of the drugs included in interaction alerts presented in Table 1, and the number of respondents who considered their action as important

	Interns (*n* = 55)		Residents (*n* = 69)		*P* value^c^
Drug	Action suggested *n* (%)	Action considered important^a^ *n* (%^b^)	Action suggested *n* (%)	Action considered important^a^ *n* (%^b^)	
Alendronic acid	12 (22)	2 (17)	15 (22)	5 (33)	1.00
Atorvastatin	11 (20)	6 (55)	21 (30)	8 (38)	0.22
Calcium	18 (33)	3 (17)	16 (23)	3 (19)	0.31
Citalopram	10 (18)	2 (20)	11 (16)	6 (55)	0.81
Clopidogrel	3 (5)	3 (100)	0	0	0.09
Hydroxyzine	24 (44)	13 (54)	50 (72)	23 (46)	0.002
Levothyroxine	11 (20)	2 (18)	14 (20)	0	1.00
Metformin	11 (20)	5 (45)	18 (26)	6 (33)	0.52
Omeprazole	39 (71)	17 (44)	57 (83)	26 (46)	0.08
Repaglinide	53 (96)	49 (92)	66 (96)	59 (89)	1.00

^a^Rating 4 or 5 on a scale from 1 = not at all important, to 5 = very important

^b^Percentage of residents that had acted on the drug

^c^Interns versus residents, whether an action was suggested

Regarding the extent to which the respondents usually access detailed information about alerted drug interactions classified as D or C, available one mouse click away, there were 95 (77%) replies. Of these, 56 (59%) respondents stated they do so for D alerts, versus 29 (31%) for C alerts (*P* < 0.001; Table 3). Of the 85 (69%) respondents who answered questions regarding whether the documentation level influenced their reading of more detailed information, 37 (43%) stated they accessed only documentation corresponding to studies on humans (levels 3 or 4), versus 18 (21%) who also accessed studies corresponding to documentation levels 0–2 (*P* = 0.003).

**Table 3:** Number (percentage) of interns and residents who stated that they access detailed drug interaction information^a^ about alerts, by classification of clinical significance and level of documentation

		Interns (*n* = 55)	Residents (*n* = 69)
Classification of clinical significance^b^	D	24 (44)	32 (46)
	C	12 (22)	17 (25)
	B	N/A	5 (7)
Level of documentation^c^	4	15 (27)	21 (30)
	3	9 (16)	17 (25)
	2	3 (5)	10 (14)
	1	2 (4)	7 (10)
	0	2 (4)	10 (14)

*N/A* not applicable

^a^Responding 4 or 5 on a scale from 1 = never, to 5 = always

^b^*D* clinically significant interaction that should be avoided, *C* clinically significant interaction that can be managed by, e.g., a dose adjustment, *B* the clinical significance is uncertain or varies

^c^4 = controlled studies in relevant populations; 3 = studies with healthy volunteers, and/ or pilot studies among patients; 2 = well-documented case reports; 1 = incomplete case reports and/or in vitro studies; and 0 = data from studies based on similar drugs

## Discussion

This study shows that nearly all physicians state that they act on one of the drugs within a drug pair alerted as a D interaction, and that 91% rate that action as important. Likewise, but to a somewhat smaller extent, drugs within drug pairs alerted as C interactions are often said to be acted upon. Whereas 59% of the respondents state that they usually access detailed information regarding alerted D interactions, only 31% do so for C alerts. The level of documentation seems to be associated with the use of the knowledge resource in a similar way; twice as many of the respondents state that they access detailed information for alerts with level 3/4 documentation, compared with other levels of documentation.

Repaglinide and clopidogrel triggered an alert classified as D, with a medical consequence described as an increased risk of hypoglycaemia. The underlying mechanism for this PK interaction is described as the inhibition of CYP2C8, the main enzyme that metabolises repaglinide, by a clopidogrel metabolite [19]. Consequently, the exposure to repaglinide may increase. Our finding that this D interaction alert was frequently acted upon is in line with a previous study that reported that the prevalence of D interactions was reduced when Janusmed was integrated into primary healthcare records [20].

Regarding repaglinide, 47% of the interns and 68% of the residents suggested an action that included a switch to another drug. Interestingly, 22% of the residents suggested a specific drug, primarily a glucagon-like peptide-1 (GLP-1) agonist, a dipeptidyl peptidase-4 (DPP-4) inhibitor, or a sodium-glucose transport protein 2 (SGLT2) inhibitor, whereas only one intern suggested a specific drug. On the other hand, 18% of the interns described that one of their actions would be to a consult a senior college or to refer to primary care for follow-up. One could speculate that these findings may illustrate differences in alert-related actions associated with the stage of career as well as the clinical setting. Interns, at the very beginning of their career and before obtaining a full licence to prescribe, may less readily make their own treatment decisions compared with residents. Furthermore, actions in the hospital setting may focus on the acute health condition necessitating in-hospital care, and other actions may not be medically prioritised.

Omeprazole was involved in four PK interaction alerts, in drug pairs including either citalopram, clopidogrel, calcium, or levothyroxine. Overall, 77% of the respondents suggested an action related to omeprazole, but only 45% rated this action as important. Of those who acted on omeprazole, 78% suggested a switch to pantoprazole, i.e., consistent with the recommendation provided by Janusmed regarding the combined use of citalopram and clopidogrel [19]. Interestingly, the action to stop omeprazole was suggested more than twice as often by residents compared with interns. Again, the divergent approach may be related both to the gradual development of professional autonomy regarding drug treatment decisions and to the clinical setting. Clearly, a switch to another proton pump inhibitor (PPI), described as less prone to enzyme inhibition than omeprazole [21], could be regarded as a minor treatment decision whereas stopping a drug may require more of the prescriber. Furthermore, as the withdrawal of a PPI has been associated with acid rebound effects [22], the primary care setting may be preferable as it allows follow-up. Another aspect worth noting is that three interns suggested to stop clopidogrel, or to switch this drug to another drug, whereas none of the residents suggested such an action. One may speculate that residents, having come further in the physician career, may be more aware of challenges related to cardio- and cerebrovascular prevention.

Omeprazole is described to interact with levothyroxine and calcium at the absorption level, with reduced uptake as the consequence. In the patient case, two respondents suggested to increase the calcium dose and 11 respondents to monitor thyroid-stimulating hormone (TSH), both actions consistent with the Janusmed recommendations. Few respondents, however, considered actions related to calcium or levothyroxine important. It could be speculated that this finding reflects that the B classification of the omeprazole/calcium alert, may contribute to the perception of low importance. In addition, the monitoring of TSH in relation to the omeprazole/levothyroxine alert has previously been reported as being in general adequate, not requiring further action [15].

In Janusmed, the hydroxyzine/citalopram alert is described as a PD interaction. Both drugs may prolong the QT interval in a dose-related manner [23, 24], and the risk could increase when two QT-prolonging agents are combined [25], which, in turn, may increase the risk of torsade de pointes. The inclination for action regarding hydroxyzine, as well as the suggested actions, differed between interns and residents; 46% of the residents suggested to stop this drug whereas only 16% of the interns suggested such an action. A range of substitute drugs were suggested by both interns and residents, including melatonin, mirtazapine, oxazepam, promethazine, and zopiclone, representing common choices in the treatment of anxiety [26]. The fact that the recommendation provided by Janusmed did not include a switch to a specific drug may have contributed to the diversity of proposed treatment strategies. Interestingly, only about half of the respondents rated their suggested action for hydroxyzine as important, although this drug is included in several sets of potentially inappropriate medications for older people [27–29].

Our findings highlight the importance of the classifications in a drug interaction knowledge resource. Physicians at the early stages of the career seem prone to read more of the extended information when an alert is classified as having “higher” clinical significance. On the one hand, these results may be considered encouraging; alerts that are likely to have a greater impact on patients seem to receive more attention. On the other hand, this finding highlights the importance of trustworthy classifications. In this context, it must be mentioned that classifications differ somewhat between drug interaction knowledge resources [30–32]. Furthermore, the level of documentation seems to guide physicians’ readiness to click for more information about a drug interaction alert. Thus, the presence of clinical studies, i.e., level 3 or 4 documentation, seems to encourage such access. Nevertheless, case reports and in vitro studies, as well as studies on similar drugs, could deserve more attention by physicians.

### Strengths and limitations

An important strength of this study is that it contributes knowledge on how physicians at early stages of their career act on drug interaction alerts and how important they consider these actions to be. Furthermore, differences between hospital interns and residents in family medicine, representing the hospital and primary care setting, are explored, as well as the importance of classifications in a knowledge resource. It may also be considered a strength that the questionnaire was anonymous. This approach allowed the respondents to provide honest unbiased replies as there was no concern for repercussions. Furthermore, the results are based on a nearly 100% response rate, an aspect of importance for generalisability. The response rate for some of the general questions concerning information access, however, was lower. Another limitation of potential importance for the external validity is that our respondents represent Swedish healthcare and the national knowledge resource, Janusmed, used in this setting. Nevertheless, this resource has apparent similarities with internationally established well-renowned resources like Lexicomp, Micromedex, and Stockley’s [33–35]. Indeed, they all provide interaction alerts that are classified regarding the clinical significance and the level of documentation, with recommendations for clinical management and references for further reading [13].

An important limitation of this study is that the fictional patient case only included one alert classified as D, making comparisons with the more numerous C alerts difficult. Another limitation is that the respondents suggested actions per drug and not per drug pair eliciting an interaction alert, thereby precluding direct linking of actions to a specific alert. Furthermore, despite the fact that the respondents were instructed verbally and in writing to state any actions taken *due to the risk of interactions*, we cannot rule out that some actions may have been suggested for other reasons, such as switching to a drug considered more effective or less prone to evoke ADRs. However, the provided actions for each drug mirror the real-life situation when physicians prescribe drug treatment – prescribing concerns specific drugs in an entire medication list, not single drug pairs, and the context of the patient is taken into account. Finally, it must be stressed that this study does not evaluate the value of decision support regarding potentially problematic drug interactions.

## Conclusion

This study shows that physicians act on drug interaction alerts with considerable variation, and that hospital interns in some respects differ from residents in family medicine, perhaps representing different stages of the career and different work settings. Recommendations for clinical management provided by the knowledge resource are quite often adhered to, and classifications of drug interaction alerts appear to guide physicians regarding whether to access more detailed information provided by the knowledge resource.

## Supplementary Information

Below is the link to the electronic supplementary material.

Supplementary file1 (PDF 260 KB)

Supplementary file2 (DOCX 21.9 KB)

Supplementary file3 (DOCX 28.7 KB)

## Data Availability

The datasets generated during the current study are available from the corresponding author upon reasonable request. The R code used for analysis is available at: https://pharm.nu/suppl/tukukino2024/
