# Supplementary material for: Pharmacotherapeutic actions related to drug interaction alerts – a questionnaire study among Swedish hospital interns and residents in family medicine
Source: Eur J Clin Pharmacol. 2024 Dec 16;81(2):301–8. doi: 10.1007/s00228-024-03785-4 (PMC11717818; doi:10.1007/s00228-024-03785-4)
Supplement: Supplementary file 1 — Supplementary file1 (PDF 260 KB) [file 228_2024_3785_MOESM1_ESM.pdf]

Online Resource 1 Questionnaire distributed in print to interns and residents

Pharmacotherapeutic actions related to drug interaction alerts – a questionnaire study among Swedish hospital interns and residents in family medicine

Carina Tukukino, Naldy Parodi López, Johan Lönnbro, Susanna M Wallerstedt, Staffan A Svensson

Comments regarding the translation

The questionnaire, in the Swedish original, exists in three versions. This was mainly due to the respondents' use of different types of electronic health record (EHR) systems, and the varying ways in how interaction alerts are presented in these. Residents were asked to choose, depending on their EHR, between one of two versions. These differed somewhat in the phrasing of questions about clicking on buttons in the medication module.

The interns, using yet another EHR, were given a third version. This also differed from that of the residents, in terms of questions related to work experience.

As the abovementioned differences are irrelevant for the present study, the translation shown here represents a unified version. Only the first and second parts of the questionnaire have been translated, the first dealing with assessment of the patient case and the second with general questions on interaction alerts. The third section, which concerns demographic data and work experience, has not been translated – its questions are straightforward and should be easy to understand from the Swedish original (linked below).

Some further notes:

1. In questions #1 and #2, three trade names of drugs were listed alongside the generic names, but these were not included in the translation.

2. The residents' questionnaires had a question #3 concerning what EHR they used (the reply alternatives being: "Asynja", "AsynjaVisph/TakeCare", "Webdoc", "Journal 4", or "Other"). All the interns used the same EHR ("Melior") and did not need this question, it was omitted also in the translation.

The original three questionnaires, as well as the interaction alert texts handed out to respondents, are available in this web folder: https://pharm.nu/suppl/tukukino2024

Online Resource 1 Questionnaire distributed in print to interns and residents

The fictional patient case that the interns and residents were to assess.

Your patient is a female 73-year-old retired shop assistant who smokes 14 cigarettes a day since many years. She has a history of orally treated type 2 diabetes, mild vascular dementia (last Mini Mental State Exam: 21/25), anxiety, osteoporosis, hypothyroidism, and dyspepsia (a gastroscopy a few months ago was normal).

[*Interns (in hospital)*] She ended up in hospital last night due to a suspected TIA and was put on clopidogrel via the emergency department ... you must now discharge her home…

[*Residents (in primary care centre)*] She has recently been hospitalised due to a suspected TIA and was put on clopidogrel …with a referral to you for follow-up. At the planned follow-up visit…

…the patient tells you that she feels fine, having recovered from the TIA, and experienced no new symptoms.

On examination, you find a pleasant woman with normal neurological status, mild ankle oedema, lungs and heart clear to auscultation, regular heart rate 76 bpm. Fasting blood glucose is 7.9 mmol/L (142 mg/dL) HbA1c 57 mmol/mol (7.4% DCCT). Weight is 82 kg, BMI 31, eGFR 56 mL/min/1.73m2, haemoglobin 12.6 g/dL, LDL 2.6 mmol/L (100 mg/dL).

The patient takes a number of drugs regularly (all oral), as well as hydroxyzine PRN.

Atorvastatin 20 mg 1+0+0
Repaglinide 2 mg 1+0+0
Metformin 500 mg 1+1+1
Calcium/D3 500 mg/400E 0+1+0
Alendronic acid 70 mg (every Sunday)
Citalopram 20 mg 1+0+0
Omeprazole 20 mg 1+0+0
Levothyroxine 75 mg 1+0+0
Clopidogrel 75 mg 1+0+0
Hydroxyzine 25mg, 1 PRN, maximum 2 per day

The medication list triggers several drug interaction alerts, see printout from Janusmed and Table 1.

[*Interns*] The patient asks you for new prescriptions and you decide to help her with this.
[*Residents*] -

Do you take any action due to the risk of interaction between the drugs?

When you answer the questions: imagine the context “a normal day at work” with time constraints, priorities and so on. Read the attached information from Janusmed as you usually would do: nothing, parts of it, or all of it – this is not a knowledge test but an attempt to estimate how a physician behaves in everyday practice.

Online Resource 1 Questionnaire distributed in print to interns and residents

Part one: A specific patient case (please refer to article's Box)

**1.** For all drugs below, either tick "no action", or any suggested action/s. If you tick "other action", describe it.	No action	Reduce dose	Stop drug	Increase dose	Other action	Comments
Atorvastatin 20 mg 1+0+0	☐	☐	☐	☐	☐	________
Repaglinide 2 mg 1+0+0	☐	☐	☐	☐	☐	________
Metformin 500 mg 1+1+1	☐	☐	☐	☐	☐	________
Calcium/D3 500 mg/400E 0+1+0	☐	☐	☐	☐	☐	________
Alendronic acid 70 mg (every Sunday)	☐	☐	☐	☐	☐	________
Citalopram 20 mg 1+0+0	☐	☐	☐	☐	☐	________
Omeprazole 20 mg 1+0+0	☐	☐	☐	☐	☐	________
Levothyroxine 75 mg 1+0+0	☐	☐	☐	☐	☐	________
Clopidogrel 75 mg 1+0+0	☐	☐	☐	☐	☐	________
Hydroxyzine 25 mg 1 PRN, maximum 2 per day	☐	☐	☐	☐	☐	________

**2.** How important were the actions you suggested in the previous question (if any). Leave blank for drugs with no suggested actions.	No action	1.Not at all important	2.	3.	4.	5. Very important
Atorvastatin 20 mg 1+0+0	☐	☐	☐	☐	☐	☐
Repaglinide 2 mg 1+0+0	☐	☐	☐	☐	☐	☐
Metformin 500 mg 1+1+1	☐	☐	☐	☐	☐	☐
Calcium/D3 500 mg/400E 0+1+0	☐	☐	☐	☐	☐	☐
Alendronic acid 70 mg (every Sunday)	☐	☐	☐	☐	☐	☐
Citalopram 20 mg 1+0+0	☐	☐	☐	☐	☐	☐
Omeprazole 20 mg 1+0+0	☐	☐	☐	☐	☐	☐
Levothyroxine 75 mg 1+0+0	☐	☐	☐	☐	☐	☐
Clopidogrel 75 mg 1+0+0	☐	☐	☐	☐	☐	☐
Hydroxyzine 25 mg 1 PRN, maximum 2 per day	☐	☐	☐	☐	☐	☐

Online Resource 1 Questionnaire distributed in print to interns and residents

Part two: Drug interaction alerts in general

		1. Never	2.	3.	4.	5. Always
**4:1** Faced with an interaction alert, how often do you click the medication module's button to read more about the interaction's medical consequences and recommendations?		☐	☐	☐	☐	☐
		1.Never click further	2.	3.	4.	5. Always click further
If you ticked anything but "never" in question 4:1: How often do you click on to read medical consequences and recommendations for alerts classified as *D-, C- and B?*	**D-classification**	☐	☐	☐	☐	☐
	**C-classification**	☐	☐	☐	☐	☐
	**B-classification**	☐	☐	☐	☐	☐
		1. Never click further	2.	3.	4.	5. Always click further
**4:2** How often do you click further, one more step, to read in-depth information (background information, cited references) concerning the interaction alert?		☐	☐	☐	☐	☐
If you ticked anything but "never" in question 4:2: How often do you click further one more step to read background/cited references for alerts classified as *D-, C- and B?*	**D-classification**	☐	☐	☐	☐	☐
	**C-classification**	☐	☐	☐	☐	☐
	**B-classification**	☐	☐	☐	☐	☐
		1. Never	2.	3.	4.	5. Always
**4:3** How often does the interaction alert's level of documentation affect if you click further to read in-depth information (background, cited references)?		☐	☐	☐	☐	☐
		1 Never click further	2.	3.	4.	5 Always click further
If you ticked anything but "never" in question 4:3: How often do you click further one more step to read background/cited references for alerts with documentation levels 0–4?	**level of documentation 0**	☐	☐	☐	☐	☐
	**level of documentation 1**	☐	☐	☐	☐	☐
	**level of documentation 2**	☐	☐	☐	☐	☐
	**level of documentation 3**	☐	☐	☐	☐	☐
	**level of documentation 4**	☐	☐	☐	☐	☐

Online Resource 1 Questionnaire distributed in print to interns and residents

	1. Not agree at all	2.	3.	4.	5. Totally agree
**5. To what extent do you agree with the following statement?**					
I get useful advice from Janusmed about how I should manage an interaction alert when prescribing drugs	◻	◻	◻	◻	◻

6. Do you have any opinions regarding drug interaction alerts in relation to your professional role?

____

____

____

____

____

____

Part three: Characteristics

Questions concerning respondents' age, gender, years of completed training, site of education and work experience (not translated).
