# Supplementary material for: Pharmacotherapeutic actions related to drug interaction alerts – a questionnaire study among Swedish hospital interns and residents in family medicine
Source: Eur J Clin Pharmacol. 2024 Dec 16;81(2):301–8. doi: 10.1007/s00228-024-03785-4 (PMC11717818; doi:10.1007/s00228-024-03785-4)
Supplement: Supplementary file 2 — Supplementary file2 (DOCX 21.9 KB) [file 228_2024_3785_MOESM2_ESM.docx]

**Pharmacotherapeutic actions related to drug interaction alerts – a questionnaire study among Swedish hospital interns and residents in family medicine**

Carina Tukukino, Naldy Parodi López, Johan Lönnbro, Susanna M Wallerstedt, Staffan A Svensson

**Table S2** Characteristics of responding interns (n=55) and residents (n=69)

|  |  | | **Interns**  **n (%)** | **Residents**  **n (%)** |
| --- | --- | --- | --- | --- |
| Age (years) | 26–30 | | 31 (56) | 10 (15) |
|  | 31–40 | | 12 (22) | 44 (64) |
|  | 41–50 | | 6 (11) | 11 (16) |
|  | 51–60 | | 0 | 4 (6) |
|  | NR | | 6 (11) | 0 |
| Gender | Woman | | 32 (58) | 33 (48) |
|  | Man | | 16 (29) | 33 (48) |
|  | NR | | 7 (13) | 3 (4) |
| Country where the medical degree was obtained | Sweden | | 54 (98) | 38 (55) |
|  | Other EU country | | 0 | 24 (35) |
|  | Outside EU | | 0 | 7 (10) |
|  | NR | | 1 (2) | 0 |
| Employment | Regular internship | | 31 (56) | N/A |
|  | Internship with time for research included | | 23 (42) | N/A |
|  | NR | | 1 (2) |  |
| Work experience | Completed time of internship | <1 months | 15 (27) | N/A |
|  |  | 1–6 months | 15 (27) | N/A |
|  |  | 0.5–1 year | 19 (35) | N/A |
|  |  | >1 year | 1 (2) | N/A |
|  |  | NR | 5 (9) | N/A |
|  | Worked as an assistant physician (before internship) | n (%) | 42 (76) | N/A |
|  |  | Median number of months (range) | 12 (2-30) | N/A |
|  | Completed years of residency in family medicine | <1 | N/A | 3 (4) |
|  |  | 1–2 | N/A | 20 (29) |
|  |  | 2–3 | N/A | 14 (20) |
|  |  | 3–4 | N/A | 22 (32) |
|  |  | >4 | N/A | 10 (15) |
|  | Already specialist in another medical specialty | | N/A | 11 (16) |

EU = European Union, N/A = not applicable, NR = not reported
