# Supplementary material for: Pharmacotherapeutic actions related to drug interaction alerts – a questionnaire study among Swedish hospital interns and residents in family medicine
Source: Eur J Clin Pharmacol. 2024 Dec 16;81(2):301–8. doi: 10.1007/s00228-024-03785-4 (PMC11717818; doi:10.1007/s00228-024-03785-4)
Supplement: Supplementary file 3 — Supplementary file3 (DOCX 28.7 KB) [file 228_2024_3785_MOESM3_ESM.docx]

**Pharmacotherapeutic actions related to drug interaction alerts – a questionnaire study among Swedish hospital interns and residents in family medicine**

Carina Tukukino, Naldy Parodi López, Johan Lönnbro, Susanna M Wallerstedt, Staffan A Svensson

**Table** The drugs included in the fictional patient’s medication list and the suggested actions by respondents for the specific drug as well as the classification of drug interaction alerts each drug appeared in.

| **Drug** | **Number of alerts by classification^a^** | | | **Number of actions by respondents (%)** | **Actions suggested by the…** | |  |
| --- | --- | --- | --- | --- | --- | --- | --- |
|  | D | C | B |  | *…interns (n=55)* | *…residents (n=69)* |  |
| **Clopidogrel** | 1 | 2 | 1 | 3 (2) | Stop drug and discuss with senior colleague (n=1)  Switch to acetylsalicylic acid (n=1)  Check indication and switch drug (n=1) | (-) |  |
| **Repaglinide** | 1 | - | - | 119 (96) | Stop drug (n=8)  Stop drug and follow up by GP (n=3)  Stop drug and consult senior colleague (n=1)  Stop drug, insulin as needed (n=1)  Stop drug temporarily (n=1)  Stop drug, SGLT2? (n=1)  Stop drug and consider switch to unknown (n=1)  Stop or decrease dose, consult senior colleague (n=1)  Switch drug to other hypoglycaemic drug (n=11)  Switch drug to unknown (n=14)  Switch drug and consult a diabetes physician (n=2)  Decrease dose (n=2)  Decrease dose or stop drug (n=2)  Decrease dose if blood glucose is monitored daily (n=1)  Consult senior colleague (n=2)  Follow up at primary care (n=1)  Probably keep the drug due to diabetes (n=1) | Stop drug (n=15)  Stop or switch drug to unknown (n=3)  Stop drug, consider SGLT2 (n=1)  Stop drug, consider metformin (n=1)  Stop drug due to clopidogrel (n=1)  Switch drug to unknown (n=18)  Switch to another hypoglycaemic drug (n=10)  Switch to GLP1/SGLT2 (n=10)  Switch to SGT2 or semaglutide (n=1)  Switch to semaglutide (n=1)  Switch to sitagliptin (n=1)  Switch to sitagliptin, monitor renal values (n=1)  Decrease dose (n=1)  Increase dose (n=1)  Other – not stated (n=1) |  |
| **Omeprazole** | - | 3 | 1 | 96 (77) | Stop drug (n=4)  Check if indication persists and consider stopping drug (n=1)  Switch to pantoprazole (n=32)  Switch drug to unknown (n=1)  Reschedule drug intake (n=1) | Stop drug (n=13)  Switch to pantoprazole (n=39)  Switch to pantoprazole? if needed (n=1)  Switch to pantoprazole and reschedule intake (n=3)  Switch to another PPI (n=1) |  |
| **Citalopram** | - | 3 | - | 21 (17) | Stop drug temporarily (n=1)  Switch to escitalopram (n=1)  Switch to escitalopram and monitor ECG (n=1)  Decrease dose (n=2)  Decrease dose and consider switch to mirtazapine (n=1)  Monitor for increased risk of bleeding (n=1)  Monitor ECG for prolonged QT interval (n=1)  Monitor ECG for prolonged QT interval and inform patient about risk citalopram- hydroxyzine (n=1)  Discuss dose and follow up (n=1) | Stop drug (n=1)  Switch to another drug (n=2)  Switch to mirtazapine (n=1)  Switch to sertraline (n=1)  Decrease dose or consider switch to sertraline  and monitor ECG (n=1)  Decrease dose (n=4)  Monitor ECG (n=1) |  |
| **Levothyroxine** | - | 2 | 1 | 25 (20) | Decrease dose (n=1)  Reschedule drug intake (n=3)  Increase dose (n=2) Monitor TSH (n=4)  Monitor TSH and adjust dose (n=1) | Decrease dose (n=1)  Reschedule drug intake (n=4)  Separate intake (n=3)  Monitor TSH (n=6) |  |
| **Calcium** | - | 2 | 1 | 34 (27) | Separate intake (n=16)  Increase dose and separate intake (n=1)  Switch drug (n=1) | Separate intake (n=15)  Increase dose and check laboratory values to adjust dose (n=1) | |
| **Alendronic acid** | - | 1 | - | 27 (22) | Separate intake (n=11)  Increase dose (n=1) | Stop drug if used >5 years (n=1)  Separate intake (n=13)  Decrease dose (n=1) | |
| **Hydroxyzine** | - | 1 | - | 74 (60) | Stop drug (n=6)  Stop drug and discuss with patient (n=2)  Stop drug, not recommended in elderly (n=1)  Switch drug to unknown (n=4)  Switch to melatonin (n=1)  Switch to propiomazine (n=1)  Switch to promethazine (n=2)  Decrease dose (n=3)  Decrease dose since risk for prolonged QT interval  together with citalopram (n=1)  Monitor ECG (n=2)  Monitor ECG for prolonged QT interval and inform patient about risk citalopram - hydroxyzine (n=1) | Stop drug (n=29)  Stop drug due to age (n=1)  Stop drug due to age and risk of QT prolongation (n=1)  Stop drug if citalopram continues or switch to sertraline (n=1)  Switch drug to unknown (n=2)  Switch to promethazine (n=4)  Switch to oxazepam (n=3)  Switch to mirtazapine (n=1)  Consider switch to zopiclone (n=1)  Decrease dose (n=2)  Monitor ECG (n=1)  Drug intake evening/night (n=1)  Check need and frequency (n=2)  Other – not stated (n=1) | |
| **Atorvastatin** | - | - | 1 | 32 (26) | Switch to rosuvastatin (n=1)  Increase dose (n=7)  Increase dose and follow up LDL by GP (n=1)  Reschedule drug intake (n=1)  Other – not stated (n=1) | Stop drug (n=1)  Switch to rosuvastatin (n=5)  Switch to rosuvastatin and monitor any drug interaction (n=1)  Increase dose (n=12)  Add ezetimibe (n=1)  Reschedule drug intake (n=1) | |
| **Metformin** | - | - | 1 | 29 (23) | Decrease dose (n=2)  Decrease dose and reschedule drug intake (n=1)  Increase dose (n=4)  Monitor laboratory values (n=3)  Follow up by GP (n=1) | Increase dose (n=17)  Monitor laboratory values (n=1) | |

^a^*Letter (classification of clinical significance)*: D *=* clinically significant interaction that should be avoided; C = clinically significant interaction that can be managed by, e.g., a dose adjustment; B = the clinical significance is uncertain or varies. (-) no action suggested, ECG = electrocardiogram, GLP = glucagon-like peptide, GP = general practitioner, LDL = low-density lipoprotein, SGLT = sodium-glucose transporter, TSH = thyroid-stimulating hormone.
